# Supplementary material for: Compositional Features of Distinct Microbiota Base on Serum Extracellular Vesicle Metagenomics Analysis in Moderate to Severe Psoriasis Patients
Source: Cells. 2021 Sep 8;10(9):2349. doi: 10.3390/cells10092349 (PMC8467001; doi:10.3390/cells10092349)
Supplement: Supplementary file 1 [file cells-10-02349-s001.zip › Figures S1,S2.pdf]

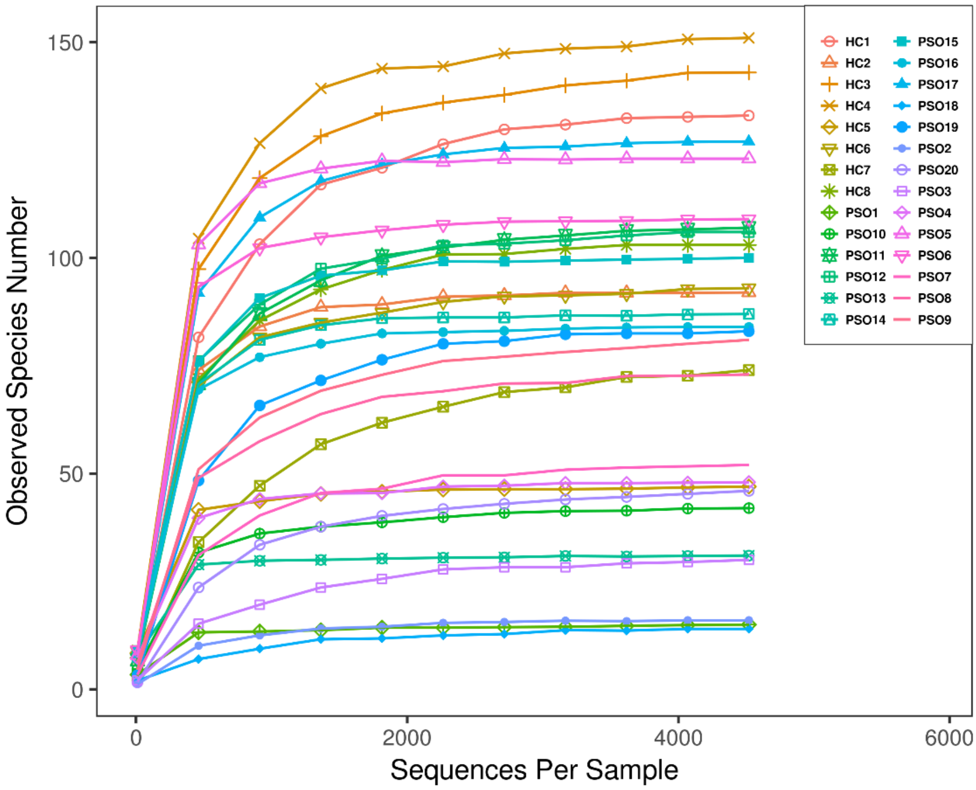


**Figure S1.** Species diversity curve - dilution curve.

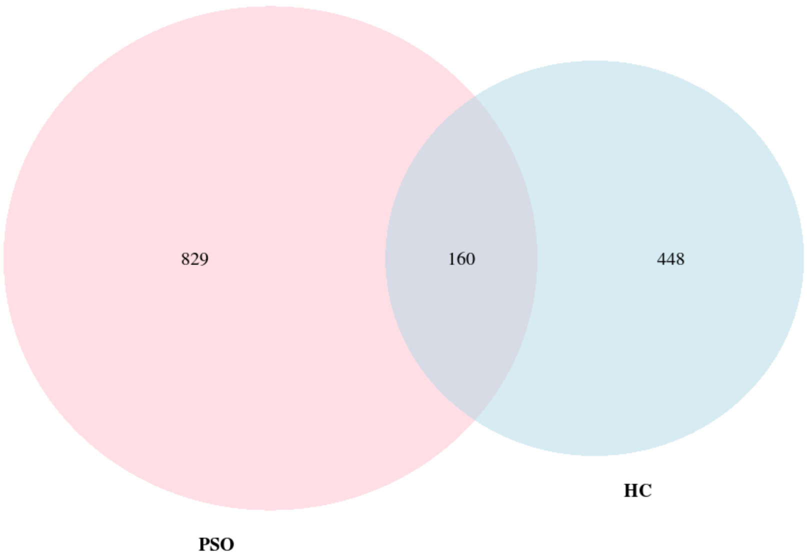


**Figure S2.** Venn diagrams of PSO group and HC group based on amplicon sequence variant (ASV) clustering.
